# CD137L-DCs, Potent Immune-Stimulators—History, Characteristics, and Perspectives

**DOI:** 10.3389/fimmu.2019.02216

**Published:** 2019-10-02

**Authors:** Qun Zeng, Yubin Zhou, Herbert Schwarz

**Affiliations:** ^1^Department of Physiology, Yong Loo Lin School of Medicine, National University of Singapore, Singapore, Singapore; ^2^Immunology Programme, Life Sciences Institute, National University of Singapore, Singapore, Singapore

**Keywords:** CD137L-DC, reverse CD137L siganling, moDC, Th1, Tc1, tumor immunotherapy

## Abstract

Dendritic cell (DC)-based immunotherapies are being explored for over 20 years and found to be very safe. Most often, granulocyte-macrophage colony-stimulating factor (GM-CSF) and interleukin-4 (IL-4)-induced monocyte-derived DCs (moDCs) are being used, which have demonstrated some life-prolonging benefit to patients of multiple tumors. However, the limited clinical response and efficacy call for the development of more potent DCs. CD137L-DC may meet this demand. CD137L-DCs are a novel type of monocyte-derived inflammatory DCs that are induced by CD137 ligand (CD137L) agonists. CD137L is expressed on the surface of antigen-presenting cells, including monocytes, and signaling of CD137L into monocytes induces their differentiation to CD137L-DCs. CD137L-DCs preferentially induce type 1 T helper (Th1) cell polarization and strong type 1 CD8^+^ T cell (Tc1) responses against tumor-associated viral antigens. The *in vitro* T cell-stimulatory capacity of CD137L-DCs is superior to that of conventional moDCs. The transcriptomic profile of CD137L-DC is highly similar to that of *in vivo* DCs at sites of inflammation. The strict activation dependence of CD137 expression and its restricted expression on activated T cells, NK cells, and vascular endothelial cells at inflammatory sites make CD137 an ideally suited signal for the induction of monocyte-derived inflammatory DCs *in vivo*. These findings and their potency encouraged a phase I clinical trial of CD137L-DCs against Epstein–Barr virus-associated nasopharyngeal carcinoma. In this review, we introduce and summarize the history, the characteristics, and the transcriptional profile of CD137L-DC, and discuss the potential development and applications of CD137L-DC.

## Introduction

The past 10 years have witnessed a renewed enthusiasm for cancer immunotherapy, prompted by the success of chimeric antigen receptor-T cells (CAR-T) and immune checkpoint inhibitors (ICI). Despite impressive therapeutic responses in some patients of certain tumors, CAR-T and ICI failed to show efficacy in most patients of the majority of cancers, especially solid cancers (1, 2). Moreover, the high frequency of severe adverse effects and the risk of breaking immune tolerance (3) by CAR-T cells or ICI emphasize that these treatments need to be carried out with caution.

A much safer immunotherapeutic approach is the use of dendritic cells (DCs). Since DCs were first described (4), substantial knowledge about the ontogeny, functions, and therapeutic applications of DCs has been accumulated, leading to the recognition that DCs exist in the form of several subsets, characterized by differences in ontogenies and functional properties (5–7). DC-based immunotherapies have been explored for two decades and are well-tolerated. Although DC-based cancer therapies has been proven to prolong the overall survival of patients, their efficacy and the clinical responses are far from satisfactory (8–10). Different strategies are being investigated to improve the efficacy, such as optimizing the tumor antigen source and loading, seeking optimal maturation methods, and the combination of DCs with ICI (11). However, first and foremost, a pivotal parameter is the source and type of DCs.

Due to the low frequency of natural DCs in blood, monocyte-derived DCs (moDCs), obtained by treating human peripheral monocytes with granulocyte-macrophage colony-stimulating factor (GM-CSF) and interleukin-4 (IL-4), are currently the most commonly used DC type in clinical trials. New methods to target DCs *in vivo* (12, 13), to enrich blood DCs in GMP facilities *ex vivo* (14, 15), or to differentiate myeloid DCs from stem cells (16, 17) have been explored. Yet the yield of DCs is limited. We have found a new type of human DC, CD137 ligand-induced DC (CD137L-DC), that is differentiated from peripheral monocytes by recombinant CD137-Fc protein or anti-CD137 ligand (CD137L) antibodies (18). Compared to the commonly used GM-CSF and IL-4-induced moDCs, CD137L-DCs have shown superior activities in inducing T cell responses (19, 20). In this review, we will give a systematic review on the development, the function, and the clinical application of this new type of DCs.

## The Discovery of CD137L-DC

CD137 (TNFRSF9, 4-1BB) is an important co-stimulatory molecule expressed strictly upon activation, predominantly on T cells, NK cells, and vascular endothelial cells (21–23). Engagement of CD137 potently costimulates T cells and induces effective anti-tumor immune responses (24–27). Two agonistic anti-CD137 antibodies (urelumab and utomilumab) have shown great potency in preclinical experiments, and are currently being tested in clinical trials (28). In CAR, the intracellular domain of CD137 delivers signals for CAR-T cell persistence and delays their exhaustion (29, 30). CD137 ligand (CD137L, TNFSF9, 4-1BBL) is expressed on all types of antigen-presenting cells (APCs), and expression levels of CD137L increase upon APC activation (31). In the 1990s, several tumor necrosis factor super family (TNFSF) members were reported to trigger reverse signals into APCs (32–34). Reverse signaling is possible when a ligand is not a soluble molecule but is expressed as a transmembrane protein on the cell surface and can transmit a signal into the cell it is expressed on. Thus, functionally, it is identical to a receptor but it is referred to as a ligand (1) due to historical reasons and/or (2) because its partner molecule is also a receptor. Hence, both interacting molecules send and receive signals, i.e., act at the same time as a receptor and ligand, thereby establishing bidirectional signaling (35).

**Figure 1:**
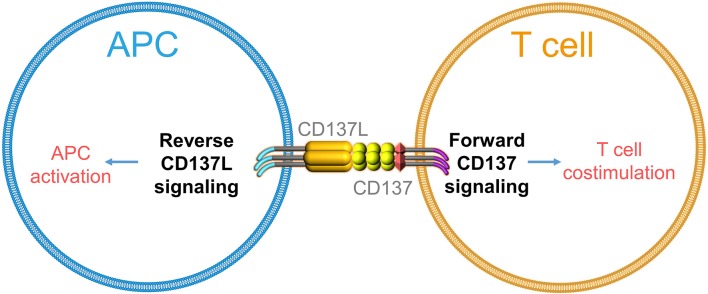
Schematic depiction of bidirectional signaling by CD137–CD137L.

Similarly, engagement of CD137L was found to cause T cell apoptosis (36) and to activate monocytes as evidenced by the induction of adherence and cytokine secretion (37). Further, immobilized CD137-Fc protein induced survival and even proliferation of monocytes, which are mainly mediated by CD137L-induced macrophage colony-stimulating factor (M-CSF) (38, 39). Reverse signaling of CD137L was further shown in monocytic cell lines (40), B cells (41), moDCs (42, 43), and myeloid DCs (44). Notably, cross-linking of CD137L matures moDCs and myeloid DCs *in vitro* as seen by the increased expression of costimulatory molecules and IL-12p40 (43, 44). Altogether, these findings demonstrate that CD137L, just like other TNFSF members, not only can deliver but also can receive a signal (Figure 1).

Human monocytes that were exposed to CD137L agonists adhered to cell culture dishes very rapidly and the resultant cells were morphologically different from resting or LPS-activated monocytes and from macrophages (37, 45). The cells exhibited extensions that were comparable with DCs but their morphology was different from DCs that were generated from monocytes by GM-CSF and IL-4 treatment. In 2009, it was found that an agonistic anti-CD137L antibody could replace GM-CSF in the differentiation of moDCs. The anti-CD137L antibody + IL-4-induced DCs stimulated stronger T cell proliferation and preferentially polarized naïve CD4^+^ T cells toward type 1 T helper (Th1) cells compared to conventional moDCs (46). However, IL-4 is not required when employing CD137-Fc protein, which alone is sufficient to induce monocyte to DC differentiation. The resulting cells, later named CD137L-DC, had an enhanced expression of the DC maturation marker CD83 and enhanced endocytosis but reduced phagocytosis and oxidative burst (18). Despite lacking the conventional moDC markers CD1a and CD209, reverse CD137L signaling-induced cells could stimulate the proliferation of naïve T cells, which is the gold standard of defining a DC, justifying their naming as a type of DC (18). The T cell-activating capability of CD137-Fc-activated monocytes is gradually acquired because during the first 24 h of differentiation, the developing CD137L-DCs induce T cell apoptosis *via* elevated reactive oxygen species. This unexpected finding may have its physiological significance in a process called infection-induced T cell attrition, where old T cells are eliminated in order to create space for the new ones with specificity for the new challenge antigens (47). Altogether, these data made it clear that reverse CD137L signaling induced the differentiation of monocytes to a new type of DC, namely, CD137L-DC.

## Species Difference in CD137L-DC

The abovementioned reverse CD137L signaling has been reported in both human and mouse. However, murine reverse CD137L signaling is very different from human reverse CD137L signaling. In murine endothelial cells, reverse CD137L signaling leads to chemokine secretion (48). In murine macrophages, reverse CD137L signaling sustains TNF secretion induced by toll-like receptor 4 (TLR4) activation (49, 50). However, reverse CD137L signaling was reported by the same group to suppress the activation of macrophages and DCs *in vivo* in mice. Kang et al. (51) found that CD137^−/−^ mice and agonistic anti-CD137 antibody-treated mice had better anti-tumor immune responses because of an increased differentiation of myeloid cells to CD103^+^ DCs and type 1 macrophages. They proposed that reverse CD137L signaling suppresses the generation of pro-inflammatory DCs and macrophages and thereby the induction of effective anti-tumor responses. This hypothesis is supported by immobilized CD137-Fc, which initiates reverse CD137L signaling, inhibiting the differentiation of CD103^+^ DCs and M1 macrophages *in vitro*. However, the more effective anti-tumor response was not seen in CD137L^−/−^ or neutralizing anti-CD137L antibody-treated mice (51). Another concern is that CD137 is also expressed on and functional in macrophages (52, 53) and DCs (41, 54). It can therefore not be ruled out that CD137 deficiency may have disturbed the equilibrium and function of endogenous DCs and macrophages.

CD137L-DCs have only been differentiated from human but not from murine monocytes. Unlike other TNFSF members that generally share 60–80% homology between mouse and human, the amino acid sequence of human CD137L protein is only 36% identical to that of murine CD137L (55). While murine monocytes also proliferate and change their morphology in response to immobilized murine CD137-Fc protein, the resulting cells are not inflammatory DCs as evidenced by the absence of DC markers and their inability to induce allogenic T cell proliferation (56). Therefore, CD137L-DC specifically refers to *in vitro* generated human CD137L-DC in this review. At present, the functions and activities of CD137L-DC have just started to be evaluated *in vivo*.

## The Superior Function of CD137L-DC

To achieve more effective anti-tumor immune responses, DCs must polarize naïve T cells preferentially to Th1 and type 1 CD8^+^ T cell (Tc1) responses. CD137L-DCs meet this requirement. CD137L-DCs enhance the subset of interferon (IFN)γ^+^ T cells, especially among the CD8^+^ cells, and no additional maturation of CD137L-DCs is required for this activity (18). CD8^+^ T cells activated by CD137L-DCs express more perforin and are more cytotoxic than T cells activated by conventional moDCs (18, 19). Most importantly, human TCR-redirected T cells are stronger activated and exert superior antigen-specific killing when activated by autologous peptide antigen-pulsed CD137L-DCs than by autologous moDCs or mature moDCs (19, 20). This superior function of CD137L-DCs has been observed with cytomegalovirus-, hepatitis B virus (HBV)-, and Epstein–Barr virus (EBV)-derived antigens. HBV and EBV are associated with various types of cancers (57, 58), implying that CD137L-DCs are a good candidate for virus-associated cancer immunotherapy. Classical DCs (cDCs) are also more potent than moDCs at activating Th1 and Tc1 responses. The direct comparison between CD137L-DCs and cDCs has not yet been done. What we know is that CD137L-DCs express low level of CD141 and no CD1c, markers for cDC1 and cDC2, respectively (59).

To achieve a successful DC-based immunotherapy, the maturation of DCs is of great importance. Although CD137L-DCs express the DC maturation marker CD83 and secrete pro-inflammatory cytokines, the cytokine levels are generally low. In order to increase the potency of CD137L-DCs, different combinations of cytokines and pattern recognition receptor agonists were compared regarding their ability to mature CD137L-DCs (19). Toll-like receptor 7/8 agonist R848 and IFNγ were found to generate the most potent mature CD137L-DCs. These mature CD137L-DCs significantly elevated the expression of CD40, CD70, CD80, CD86, CD137L, and HLA-DR (19, 60). Accordingly, mature CD137L-DCs more strongly enhance the proliferation and the percentage of IFNγ^+^ T cells (19).

**Figure 2:**
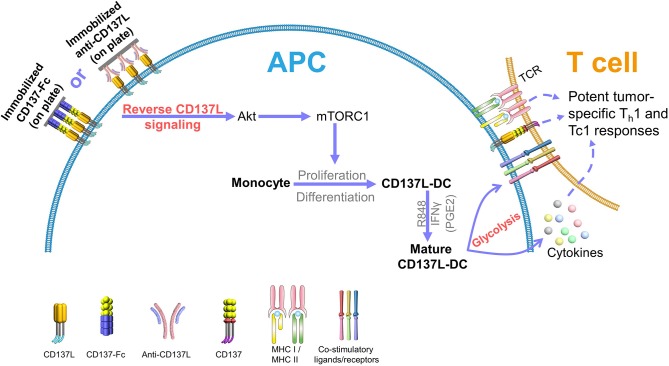
CD137L-DCs induce potent Th1 and Tc1 responses against tumors. Reverse CD137L signaling activates monocytes and induces the proliferation and the differentiation of monocytes to CD137L-DCs. R848 and IFNγ (and PGE2) cause maturation of CD137L-DCs. Mature CD137L-DCs are characterized by a high Akt-mediated glycolysis rate, which is important for the inflammatory properties of CD137L-DCs. Mature CD137L-DCs preferentially induce Th1 and Tc1 polarization in T cells, leading to strong immune responses against virus-associated tumor cells, making them a promising candidate for virus-associated cancer immunotherapy.

The ability of DCs to migrate to the lymph nodes is another pivotal factor for a successful DC-based therapy (10, 11). To boost the expression of CCR7, and thus the migratory capacity of mature CD137L-DCs, prostaglandin E2 (PGE2) was included in the maturation cocktail (61). Even though PGE2 is known to decrease the secretion of IL-12 by DCs (62), the T cell-activating capability of mature CD137L-DCs is not significantly impaired by PGE2 (20). CD8^+^ T cells activated by PGE2, R848, and IFNγ-matured CD137L-DCs are more cytotoxic, less exhausted, and metabolically more active than CD8^+^ T cells activated by mature moDCs (20). A schematic summary of CD137L-DC functions is shown in Figure 2.

Based on these findings, we started a phase I clinical trial (NCT03282617) with CD137L-DCs to treat nasopharyngeal carcinoma (NPC). The CD137L-DCs are generated from patients' monocytes; matured with R848, IFNγ, and PGE2; and pulsed with peptide pools of EBV antigens. Currently, data are available on 10 patients and no immune*-*related adverse events have been observed, demonstrating an excellent safety profile of CD137L-DCs.

## Characteristics and Physiological Relevance of CD137L-DC

The most commonly employed step between *in vitro* studies and clinical trials is the use of animal models, generally murine models. Demonstrating *in vivo* proof of principle of CD137L-DCs in murine tumor models was hindered by the above-described species difference in the molecular structure and in reverse CD137L signaling between mouse and human.

In order to investigate the *in vivo* relevance of CD137L-DC, Harfuddin et al. acquired the transcriptome of CD137L-DCs and compared it to the transcriptomes of different types of *in vitro*-generated human myeloid cells and *in vivo* inflammatory macrophages and DCs. By using hierarchical clustering and gene enrichment analysis, it was found that the gene signature of CD137L-DC is distinct from that of other myeloid cells, but is most similar to that of immature moDC and macrophages. Notably, the transcriptome of CD137L-DCs is enriched for the gene signatures of human inflammatory DCs and BDCA1^+^ DCs as they occur *in vivo* at sites of inflammation (59), indicating CD137L-DCs may exist under inflammatory conditions *in vivo* in man.

Further support for this hypothesis comes from the presence of CD137 in blood vessels at sites of inflammation and the involvement of the CD137–CD137L system in monocyte recruitment into inflamed tissues. CD137 expression is induced on vascular endothelial cells by TNF, and CD137 on the vascular endothelial cells strengthens intercellular adhesion molecule 1 and lymphocyte function-associated antigen 1-mediated adhesion of monocytes. In *in vitro* systems, CD137 attracts monocytes to infiltrate into spheroids and matrigels (63, 64). Therefore, circulating monocytes that are recruited to sites of inflammation *via* CD137 on vascular endothelial cells would receive a CD137L signal during the recruitment that may initiate their CD137L-DC differentiation. Further CD137L signaling would be induced by CD137-expressing leukocytes at the site of inflammation.

These data support the concept that reverse CD137L signaling in circulating monocytes may contribute to the physiological generation of inflammatory DCs in man. After all, CD137 expression is strictly activation-dependent and only found at sites of inflammation. Expression of CD137L is enhanced upon APC activation. This restricted expression of CD137 would confine the generation of CD137L-DCs to sites of inflammation.

The transcriptome profile of human CD137L-DC has generated additional interesting information. For example, CD137L-DCs strongly adhere to the plastic cell culture dishes. This feature finds its explanation in the gene ontology enrichment analysis. Compared to both immature and mature moDCs, CD137L-DCs express 22 genes involved in cell adhesion at more than 2-fold higher levels (59).

Another property of CD137L-DCs that was identified by the transcriptome analysis is their metabolism. Metabolic reprogramming is being increasingly appreciated as an important driving force of immune cell activation and effective immune responses (65). Our recent data demonstrate that Akt-driven glycolysis contributes to the superior function of CD137L-DCs. Compared to GM-CSF and IL-4-generated moDCs, CD137L-DCs show a significantly higher basal glycolysis rate and glycolytic capacity due to the elevated activation of the phosphoinositide 3-kinase (PI3K)–Akt–mechanistic target of rapamycin complex 1 (mTORC1) pathway. This higher rate of glycolysis is important not only for the maturation but also for the sustained activation of CD137L-DCs. The inhibition of the PI3K or Akt recapitulates the inhibition of CD137L-DC by suppressed glycolysis (Figure 2). In contrast to the flux of glycolysis intermediates into lipid synthesis in murine bone marrow-derived DCs (66), the higher glycolysis of mature CD137L-DCs leads to an increase in succinate and serine, which are known metabolites that regulate inflammation (60).

## Reverse CD137L Signaling Mechanism

An interesting aspect is the signal transduction mechanism employed by CD137L. In human primary monocytes and THP-1 cells, protein tyrosine kinases, mitogen-activated protein kinase (MAPK) kinase, p38 MAPK, extracellular regulated protein kinases 1/2, PI3K-Akt-mTORC1, and protein kinase A are activated by recombinant CD137-Fc protein (60, 67). mTORC1 is critical for the differentiation of CD137L-DCs from monocytes (60). Interestingly, CD137L associates with several cell surface receptors with well-characterized signal transduction cascades. In human cells, CD137L associates with TNFRI, which is required for CD137L-induced cell adhesion, CD14 expression, and IL-8 production (68). In murine macrophages, the association of murine CD137L with TLR4 is required for TLR4-induced activation of the transcription factors cAMP response element binding protein (CREB) and CCAAT-enhancer-binding proteins (C/EBP), and for sustained TNF secretion by murine peritoneal macrophages (49). The murine TLR4–CD137L complex associates with toll–interleukin-1 receptor domain-containing adaptor protein (TIRAP), interleukin-1 receptor-associated kinase-like 2 (IRAK2), TNF receptor–associated factor 6 (TRAF6), transforming growth factor–b–activated kinase 1 (TAK1), and TAK-binding protein 1 (TAB1) to form a larger signaling complex (50). Further, CD137L associates with transmembrane protein 126A (TMEM126A), and knockdown of TMEM126A in murine macrophages prevents the induction of tyrosine phosphorylation and the secretion of M-CSF, IL-1β, and Tenascin C (69). It is currently not known whether these associations are species-specific or apply similarly to human and mouse. Nevertheless, the common theme from all these studies is that CD137L seems not to signal by itself, but to be part of a larger signaling complex.

On reverse CD137L signaling, there is an entirely different mechanism to consider. Upon cell–cell interaction of CD137- and CD137L-expressing cells, human CD137 gets transferred to the CD137L-expressing cells by trogocytosis and then forms a complex with CD137L, which gets internalized and degraded *via* the proteasome (70–72). Whether this process is involved in mediating reverse CD137L signaling has not yet been addressed.

## Future Perspectives

CD137L-DCs are an attractive candidate for cancer immunotherapy as CD137L-DCs are more potent at inducing and strengthening Th1 and Tc1 responses than the conventional GM-CSF and IL-4-induced moDCs. CD137L-DCs are showing an excellent safety profile in NPC patients. The evaluation of their immunological efficacy and clinical usefulness is pending. Apart from NPC, other virus-associated malignancies could also be targeted by CD137L-DCs (19, 20) such as the EBV-associated Hodgkin lymphoma and HBV-associated hepatocellular carcinoma (58). How to further enhance the efficacy of CD137L-DCs will be a topic of future research. Despite the addition of PGE2 to increase the mobility of DCs, Davignon et al. have shown that most DCs die at the site of injection (73). Improving the homing of functional DCs to the lymph nodes remains a challenge. Preconditioning the injection site may be one way to improve the efficacy of CD137L-DCs (10). Additionally, the combination of CD137L-DCs with ICI could be an approach to overcome the suppressive tumor microenvironment and boost the activation of T cells (74, 75).

An issue that a CD137L-DC therapy shares with most DC therapies and personalized therapies in general is the high cost of generating the cells. Monocytes need to be harvested and converted to CD137L-DC for each patient individually under good manufacturing practice conditions. It would be a huge advantage if an approach could be developed that enables the *in vivo* generation of CD137L-DC in patients, just like how inflammatory monocyte-derived DCs are generated at the site of inflammation, when and where they are needed. For instance, controlled and site-specific CD137L agonist delivery to monocytes may fulfill this requirement and mimic the *in vivo* generation of inflammatory monocyte-derived DCs. Practically, liposome- or exosome-mediated CD137 gene/protein delivery could be suited for this task. Nevertheless, a comprehensive understanding of the mechanism of CD137L-DC differentiation is necessary for the generation of CD137L-DCs *in vivo*. CD137L likely forms a complex with other signaling mediators, and this complex formation may be necessary to induce the differentiation of CD137L-DC. A more detailed investigation of the molecules and signaling pathways leading to CD137L-DC differentiation will surely augment our understanding of the possible roles of CD137L-DCs under inflammatory conditions such as infection, autoimmune disease, and antibody treatment-induced inflammation.

CD137L-DCs have been studied for 10 years. Transcriptomic data indicate them to be close *in vitro* counterparts to *in vivo* monocyte-derived inflammatory DCs. Due to their potent immune-stimulatory activity, CD137L-DCs are being developed for cancer immunotherapy.

## Author Contributions

QZ, YZ, and HS organized, wrote, and edited the manuscript. Figures were drawn by QZ and edited by YZ and HS.

### Conflict of Interest

The authors declare that the research was conducted in the absence of any commercial or financial relationships that could be construed as a potential conflict of interest.

## Abbreviations

CD137L-DC: CD137 ligand-induced DC
DC: dendritic cell
ICI: immune checkpoint inhibitors
moDC: monocyte-derived DC.
